# S101. CLINICAL FACTORS ASSOCIATED WITH CANNABIS USE IN A CHILEAN SAMPLE OF FIRST EPISODE PSYCHOSIS PATIENTS

**DOI:** 10.1093/schbul/sby018.888

**Published:** 2018-04-01

**Authors:** Barbara Iruretagoyena, Nicolas Crossley, Alfonso Gonzalez-Valderrama, Cristian Mena, Carmen Castañeda, Camila Diaz, Juan Undurraga

**Affiliations:** 1 Universidad Católica de Chile; 2 J. Horwitz Psychiatric Institute

## Abstract

**Background:**

Cannabis has been associated with higher risk to develop psychosis and worse long-term outcomes. Cannabis use in 2016 is estimated to be 11.3% in Chile in 2016 (SENDA 2016). As in many other countries, cannabis use has steadily increased during last 20 years, across all socioeconomic groups, but especially in men and in the group aged 12 to 25 years old. At the same time, 5.7% subjects declared using high potency cannabis. We here examined the frequency of cannabis use in patients with a first episode psychosis in Chile. We also sought to identify etiologic factors associated with cannabis use, as well as its impact on clinical and functional status.

**Methods:**

We performed a cross-sectional study on patients from an outpatient Early Intervention in Psychosis unit in Chile. Data included sociodemographic characteristics, cannabis and other substance use, and standardized clinical and functional status. FAST (Functional Assessment Short Test), SS-DSM5 (Symptom Severity Scale of the DSM5 for Schizophrenia), CUPIT (Cannabis use Problem Identification Scale) and MG (Morinsky Green Adherence Questionnaire) were applied to the participants.

**Results:**

We included 80 patients, of which 23.8% used cannabis during the previous year. 47.3% of cannabis users had used cannabis with high THC concentration. 63.2% of consumers had a moderate-high score in the CUPIT scale, indicating a high prevalence of risk consumption and use disorder. Regarding variables related with cannabis use, correlation analysis showed a significant relationship with alcohol use (p<0.001), drug use (p<0.001) and duration of untreated psychosis (p=0.039, all corrected for multiple analysis.). Multivariate regression analyses including these variables along with gender and age, only showed relation with drug use (p=0.031, OR 7.94 (1.21–51.91).

Regarding cannabis use and clinical and functional outcomes, correlation analysis showed association with adherence problems as reported by physician (p= 0.026) and the Morinsky Green Adherence Questionnaire (p= 0.031). Results showed an OR of 5.23 (1.38–19.76) for adherence problems and OR 0.2 (0.05–0.75) for Morinsky Green Adherence Questionnaire reporting good adherence. There was no effect in treatment resistance, FAST score, SS-DSM5 global, cognitive or negative score.

**Discussion:**

The percentage of cannabis use in this first episode psychosis sample is high, with a large subgroup using high potency THC cannabis. Cannabis use was associated to other drug use and to treatment adherence problems reported by physician and patient. This shows the importance of substance use treatment in first episode programs.

